# The impact of post-translational modifications and subcellular localization on NLRP3 inflammasome activation: A systematic review

**DOI:** 10.1186/s12964-025-02426-2

**Published:** 2025-10-09

**Authors:** Shuchi Zhang, Muhammad Usman, Qianxi Wu, Yingjie Gao, Lifeng Fu, Maoping Chu, Chang Jia

**Affiliations:** 1 Pediatric Research Institute, The Second Affiliated Hospital and Yuying Children’s Hospital of Wenzhou Medical University, Wenzhou, 325027 Zhejiang China; 2 Children’s Heart Center, The Second Affiliated Hospital and Yuying Children’s Hospital of Wenzhou Medical University, Wenzhou, 325027 Zhejiang China; 3 Key Laboratory of Structural Malformations in Childern of Zhejiang Province, No. 109, Xueyuan West Road, Lucheng District, Wenzhou, 325027 Zhejiang China

**Keywords:** NLRP3, Post-translational modifications, Golgi, Microtubule-organizing center

## Abstract

**Background:**

The NOD-like receptor pyrin domain-containing 3 (NLRP3) inflammasome is crucial for innate immunity. However, its uncontrolled and dysregulated activation may cause various inflammatory and autoimmune disorders. Therefore, tight regulation of NLRP3 inflammasome is necessary. Numerous post-translational modifications (PTMs) are reported to play a critical role in regulating NLRP3 inflammasome activation, including (de-)phosphorylation, (de-)ubiquitination, (de-)SUMOylation, (de-)palmitoylation, (de-)acetylation, deglutathionylation, ISGylation, S-nitrosylation, and alkylation. In addition, the subcellular localization of NLRP3, involving endoplasmic reticulum (ER), mitochondria, Golgi, endosomes, and the microtubule-organizing center (MTOC), is also closely related to inflammasome assembly and activation.

**Aims of review:**

This review first describes the effects of recently found PTMs on NLRP3 inflammasome activation. Furthermore, the stage at which PTMs occur is elucidated in detail while previous reviews do not distinguish it very clearly. In addition, based on the subcellular distribution of NLRP3, this review proposes a novel spatiotemporal activation of this inflammasome.

**Key scientific concepts of review:**

This review highlights the innovative findings about the effects of PTMs and localization on NLRP3 inflammasome, which enrich the regulatory networks of this inflammasome and offer references for potential clinical translation in the future.

**Graphical abstract:**

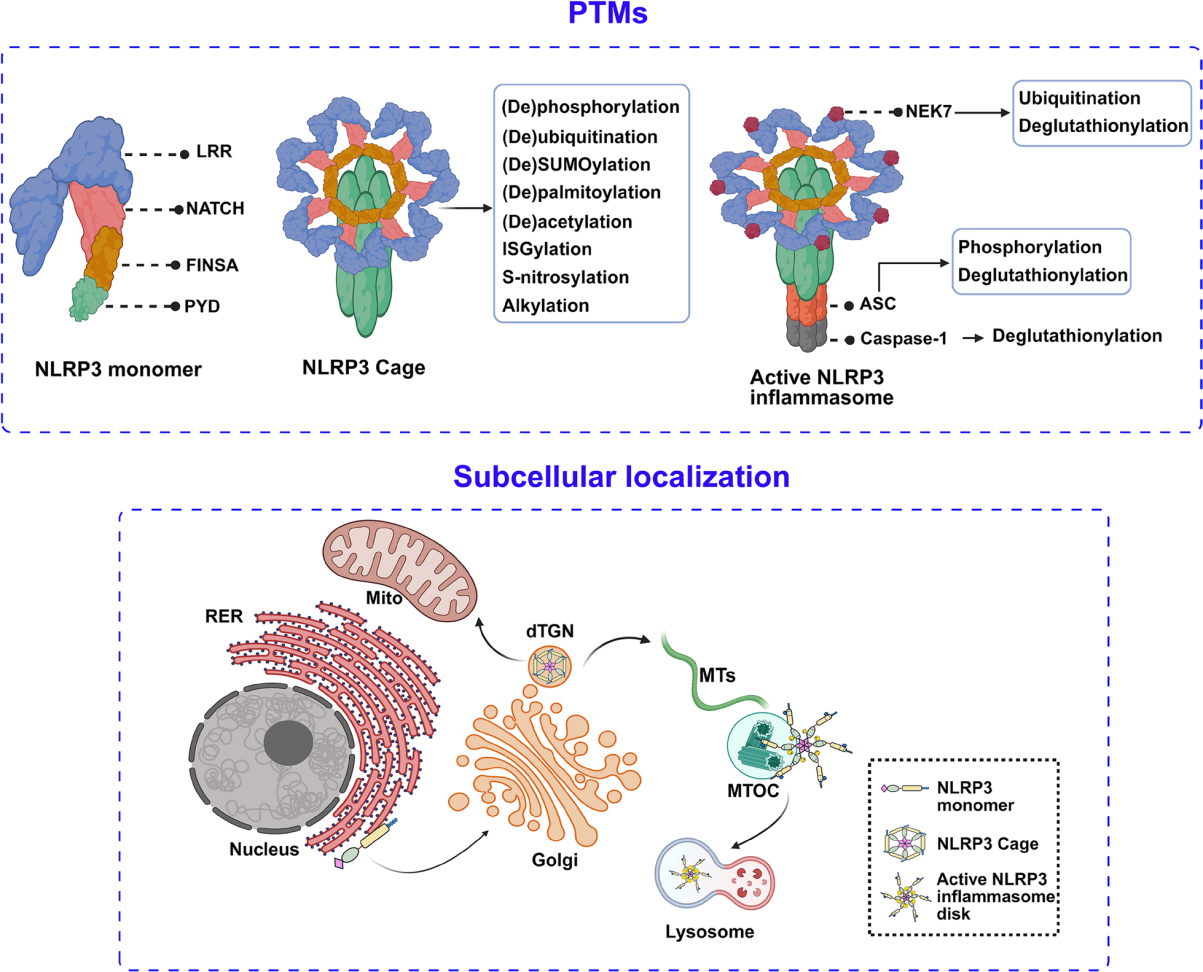

## Introduction

The NOD-like receptor pyrin domain-containing 3 (NLRP3) inflammasome is a multiprotein complex composed of NLRP3, ASC (apoptosis-associated speck-like protein containing a caspase recruitment domain), and Caspase-1 [1] or Caspase-8 [2]. Within this complex, NLRP3 consists of an N-terminal pyrin domain (PYD), a central nucleotide-binding NAIP, CIITA, HET-E, and TP1 (NACHT) domain, and a C-terminal leucine-rich repeat (LRR) domain. Among these domains, the PYD of NLRP3 facilitates interaction with the PYD of ASC, the NACHT domain’s ATPase activity drives NLRP3 self-oligomerization, and the LRR domain mediates its association with the Nima-related kinase 7 (NEK7) [3, 4]. Of course, the binding of LRR with NEK7 is redundant especially in human cell lines when other priming pathways exist [5, 6]. The inflammasome assembly is triggered by the activation of NLRP3 upon external or endogenous danger signals, which requires controlled homo-oligomerization of NLRP3 and the stepwise recruitment of ASC and Caspase-1. The fully assembled NLRP3 inflammasome activates Caspase-1, which then cleaves pro-forms of IL-1β and IL-18 and gasdermin D (GSDMD) during pyroptosis. The assembly process of NLRP3 inflammasome has been described in the previous reviews [7, 8]. It is well established that two signals, Signal 1 (priming) and Signal 2 (activation), are required and imperative for the assembly and activation of NLRP3 inflammasome and IL-1β maturation.

Priming is commonly triggered by a variety of pattern recognition receptors (PRRs), such as Toll-like receptor 4 (TLR4), alongside signaling pathways involving tumor necrosis factor (TNF), sphingosine 1-phosphate (S1P)/S1P receptor (S1PR), adenosine diphosphate (ADP)/P2Y12, or α-synuclein/CD36. These pathways converge to activate nuclear factor κB (NF-κB), resulting in the transcriptional upregulation of NLRP3, pro-IL-1β, and pro-IL-18 [9, 10]. Numerous PRR agonists also induce post-translational priming of NLRP3 through modifications, such as (de-)phosphorylation, (de-)ubiquitylation, and others, which make NLRP3 receptive to activation stimuli [3]. NLRP3 inflammasome activation (Signal 2), which contributes to the assembly of the protein complex, can be elicited by a wide range of microbial and non-microbial danger signals, including fungal, bacterial, and viral pathogens, as well as ATP, pore-forming toxins, and other particular substances [1]. Most of these signals commonly converge on K + efflux, either independently or in conjunction with lysosomal damage, mitochondrial dysfunction and reactive oxygen species (ROS) production, ultimately leading to NLRP3 inflammasome activation [10–12].

## The post-translational modifications (PTMs) of NLRP3 inflammasome

During the two phases, numerous proteins participate in regulating post-translational modifications (PTMs) of NLRP3 to modulate inflammasome activation or inhibition. In the following section, we summarize recent advances related to PTMs of NLRP3 that are associated with Signal 1 and/or Signal 2 (Table 1).

**Table 1:** Regulation of NLRP3 inflammasome activation through PTMs

PTMs	Enzymes or Molecules	Interacting Protein	Regulation of inflammasome	PTM sites	Phase	Refs
Phosphorylation	BTK	ASC	Positive	H: Y146; M: Y144	Priming	[16]
		NLRP3		H: Y136, Y140, Y143, Y168; M: Y132, Y136, Y146	Activation	[19]
	AKT	NLRP3		H: S5; M: S3	Priming	[17]
	PKG1	NLRP3		H: S448/449; M: S444/S445	Priming	[20]
	LATS1/2	NLRP3		H: S265; M: S261	Activation	[40]
	PLK4	NEK7	Negative	H: S204; M: S204	Activation	[109]
Dephosphorylation	PP2A	NLRP3	Positive	H: S5; M: S3	Priming	[18]
	PTPN11			H: Y861; M: Y858	Activation	[21]
	PTPN22			H: Y861; M: Y858	Activation	[22]
	Psoralen		Negative	H: S658; M: S656	Activation	[23]
Ubiquitination	TRIM24	NLRP3	Negative	Unknown	Priming	[24]
	Gp78			Unknown	Activation	[27]
	A20	NEK7	Negative	H: K189, K293; M: K189, K293	Activation	[28]
	TRIM50	NLRP3	Positive	Unknown	Priming, Activation	[25]
	MARCH5			H: K324, K430; M: K320, K426	Activation	[26]
	SCF-FBXL2	NLRP3	Negative	H: K689: M: K687	Priming	[30]
	Cullin1			H: K689: M: K687	Priming	[31]
Deubiquitination	OTUD6A	NLRP3	Positive	H: K430, K689; M: K426; K687	Priming	[32]
	YOD1	NLRP3	Negative	Unknown	Priming	[29]
	MARCH7			Unknown	Priming	[34]
SUMOylation	TRIM28	NLRP3	Positive	Unknown	Priming	[36]
	UBC9			H: K204; M: K200	Activation	[38]
	MAPL	NLRP3	Negative	H: K689: M: K687	Priming	[37]
DeSUMOylation	SENP6	NLRP3	Positive	Unknown	Activation	[37]
	SENP7			Unknown	Activation	[37]
Palmitoylation	ZDHHC1	NLRP3	Positive	H: C130, C958; M: C126, C955	Priming, Activation	[40]
	ZDHHC5			H: C837, C838; M: C834, C835	Priming	[45]
	ZDHHC7			H: C130, C261; M: C126	Resting; Priming; Activation	[46, 47]
	ZDHHC17			H: C419; M: C417	Activation	[48]
	FASN			H: C901; M: C898	Priming	[52]
	ZDHHC12	NLRP3	Negative	H: C844; M: C841	Activation	[43, 44]
Depalmitoylation	PPT1	NLRP3	Negative	H: C8; M: C6	Priming	[51]
	ABHD17A			H: C837, C838; M: C834, C835	Priming	[45]
	ABHD13			H: C130; C261; M: C126	Priming	[47]
Acetylation	Tau	NLRP3	Positive	H: K23, K24, K26; M: K21, K22, K24	Priming	[53]
	KAT5			H: K26; M: K24	Activation	[54]
	KAT2B			Unknown	Priming	[55]
Deacetylation	HDAC9	NLRP3	Positive	Unknown	Activation	[56, 57]
	HDAC10	NLRP3	Negative	Unknown	Priming	[58]
	SIRT2			H: K23, K24; M: K21, K22	Priming	[59]
Deglutathionylation	GSTO1	NEK7	Positive	H: C253; M; C253	Activation	[61]
	GSTO1	ASC		H: C173; M: C171	Activation	[62]
	GSTM1	Caspase-1	Negative	Unknown	Activation	[63]
ISGylation	HERC5/6	NLRP3	Positive	H: K799; M: K796	Priming	[64]
S-nitrosylation	SNAP	NLRP3	Negative	unknown	Activation	[67]
Alkylation	itaconate	NLRP3	Negative	M: C548	Activation	[77]

### Phosphorylation and dephosphorylation of NLRP3 inflammasome

Several kinases and phosphatases are involved in phosphorylation or dephosphorylation of NLRP3. Protein kinases phosphorylate NLRP3 by transferring phosphate groups to its serine, threonine, or tyrosine residues [13], while phosphatases mediate the hydrolysis of these phosphate groups to yield dephosphorylated NLRP3. Previous reviews have expounded the roles of the involved protein kinases (JNK1, MINK1, AKT, BKT, PKD, PKA, EphA2, Pyk2, Syk and PAK1) and phosphatases (PP2A, PTEN, and PTPN22) in modulating NLRP3 inflammasome activation during the priming (Signal 1) and/or activation (Signal 2) phases [9, 14, 15]. Interestingly, Bruton’s tyrosine kinase (BTK) is reported to play dual and opposite roles in the priming phase of NLRP3 inflammasome activation. One investigation revealed that BTK is activated in infiltrating macrophages in the infarct area, which enhances NLRP3 inflammasome activation by phosphorylating ASC at Tyr144 and physically interacting with both NLRP3 and ASC, thereby amplifying post-stroke inflammation [16]. In contrast, another literature disclosed that bone marrow–derived macrophages (BMDMs) from BTK-deficient mice or monocytes from patients with X-linked agammaglobulinemia (XLA) exhibited enhanced NLRP3 inflammasome activity. BTK inhibits NLRP3 oligomerization during priming phase through maintaining NLRP3 phosphorylation at human Ser5 (mouse Ser3), a residue located within the PYD interaction interface, and can be phosphorylated by AKT [17]. This sustained phosphorylation finally suppresses NLRP3 inflammasome assembly by disrupting charge–charge interactions between PYD domains. Mechanistically, BTK exerts this inhibitory effect by suppressing protein phosphatase 2 A (PP2A), which normally dephosphorylates Ser5. Consequently, sustained phosphorylation at this site prevents aberrant NLRP3 activation [18]. These studies manifest the divergent effects of BTK on different NLRP3 inflammasome components. BTK-mediated ASC phosphorylation at Tyr144 facilitates activation while BTK-maintained NLRP3 phosphorylation at human Ser5 (mouse Ser3) hinders it. However, during the activation phase, BTK promotes NLRP3 inflammasome activation. BTK phosphorylates NLRP3 at four tyrosine residues (Y136, Y140, Y143, and Y168) within the PYD-NACHT linker region in response to nigericin or monosodium urate (MSU) stimulation. This phosphorylation modifies the charge of the polybasic (PB) region peptide sequences, facilitating NLRP3 binding to organelle phosphoinositides and inflammasome nucleation. Consequently, NLRP3 translocates from intact Golgi membranes to dispersed Golgi fragments, facilitating its oligomerization, ASC polymerization, inflammasome assembly, and IL-1β release. Thus, BTK is regarded as a multifunctional regulator of NLRP3 inflammasome activation [19].

New studies found that Phosphoglycerate kinase 1 (PGK1), PTPN11, and Psoralen are involved in regulating the (de)phosphorylation of NLRP3. Ma et al. revealed that PGK1, functioning as a kinase, plays a direct role in modulating NLRP3 inflammasome activation in response to LPS through mechanisms unrelated to glycolysis. Upon LPS stimulation, CK2 phosphorylates PGK1 at S271, which in turn allows PGK1 to phosphorylate NLRP3 at S448/449 and facilitate the recruitment of USP14 to remove ubiquitin modifications, thereby promoting inflammatory responses [20]. Wang et al. confirmed that circGTF2H2C is significantly elevated in spinal cord injury (SCI) tissues, which increases the expression of IL-1β and IL-18 by competitively binding miR-1323 to upregulate PTPN11, a phosphatase capable of dephosphorylating NLRP3 at Tyr861, ultimately contributing to post-SCI inflammation [21]. The dephosphorylation of NLRP3 at Tyr861 can also be mediated by PTPN22 [22]. The natural product Psoralen mediates the dephosphorylation of NLRP3 at Ser658, inactivates the inflammasome, and alleviates neuroinflammation in the mouse model of Parkinson’s disease [23].

### Ubiquitination and deubiquitination of NLRP3 inflammasome

Ubiquitination is a Major PTM of proteins in all eukaryotes by conjugation of ubiquitin with targeted proteins. During this process, ubiquitin binds to the folded target protein, forming poly-ubiquitinated proteins which are subsequently Directed to 26 S proteasome. There the proteins are unfolded, dissociated from ubiquitin, and subsequently subjected to proteolysis, yielding short peptides, and preventing the accumulation of abnormal and redundant proteins within the cell. Ubiquitin (Ub) is one of the highly conserved proteins, which contains seven lysine residues (K6, K11, K27, K29, K33, K48 and K63), and is commonly attached to substrate proteins through lysine-directed isopeptide bonds. The ubiquitination process is catalyzed by three enzymes, including ubiquitin-activating enzymes (E1), ubiquitin-conjugating enzymes (E2), and ubiquitin-ligating enzymes (E3). Conversely, deubiquitinases are responsible for removing conjugated ubiquitin from the substrates. Previous reviews have elaborated that several proteins participate in the ubiquitination or deubiquitination processes of NLRP3 inflammasome components, including ubiquitination-related proteins (MARCH7, RNF125, Cbl-b, TRIM31, ARIH2, TRIM65, Pellino-2, Cullin1, Ubc13, β-TrCP1, HUWE1, and SCF-FBXL2), and deubiquitination-associated proteins (BRCC3, STING, UAF1, UCHL5, STAMBP, TRAF3, ABRO1, Peli1, USP7, USP8, and USP47) [9, 14, 15]. Recent investigations have unveiled additional proteins involved in these processes, including E3 ubiquitin ligases (TRIM24, TRIM50, MARCH5, and gp78/Insig-1), the ubiquitin-editing enzyme A20, and deubiquitin-associated proteins (YOD1, OTUD6A, UPS22 and USP5).

Tripartite motif-containing 24 (TRIM24), as a RING-type E3 ubiquitin ligase that mediates target protein ubiquitination, is downregulated in ectopic endometrium of endometriosis compared with normal endometrium. Further studies demonstrated that TRIM24 interacts with NLRP3 to promote the ubiquitination of NLRP3 during the priming phase, thereby exerting a negative regulatory effect on NLRP3/Caspase-1/IL-1β-mediated pyroptosis and migration in human endometrial stromal cells (hESC) [24]. TRIM50, another member of the tripartite motif family, modulates NLRP3 inflammasome activation in THP1 cells and mouse peritoneal macrophages (PMs) upon LPS and ATP stimulation. During Signal 1, it mitigates NLRP3 ubiquitination and maintains the stability of this protein. In the activation process, it enhances direct interaction with NLRP3 through its RING domain and triggers NLRP3 oligomerization via its coiled-coil domain, ultimately promoting inflammasome activation [25]. MARCH5 is an E3 ubiquitin ligase localized on the mitochondrial outer membrane. It maintains mitochondrial homeostasis by linking ubiquitin to target proteins to eliminate protein aggregates accumulated on the mitochondria. During the activation phase mediated by ATP or nigericin, MARCH5 in THP1 cells and BMDMs interacts with the NACHT domain of NLRP3 and drives K27-linked polyubiquitination at residues K324 and K430. This modification is necessary for NEK7 binding, fostering the formation of NEK7-NLRP3 multimeric oligomers, which promotes ASC speck formation and IL-1β release [26]. Gp78, a membrane-bound E3 Ligase that usually interacts with the ER membrane protein insulin-induced gene 1 (Insig-1), plays critical roles in diverse biological events. Although gp78 deficiency in macrophages has little effect on LPS-induced NF-κB and MAPK activation, it profoundly influences the activation phase. Gp78 engages with NLRP3 through its CUE (coupling of ubiquitin to ER degradation) domain, orchestrating mixed ubiquitination (encompassing K48-, K64-, K6-, and K11-linked chains) on NLRP3. This process curtails NLRP3 oligomerization and mitochondrial translocation, thereby blocking inflammasome assembly and activation. Notably, Insig-1 is indispensable for the gp78–NLRP3 interaction and ubiquitination process. Genetic ablation of either gp78 or Insig-1 results in hyperactivation of the NLRP3 inflammasome [27]. A20 is a ubiquitin-editing enzyme, deficiency of which increases the protein and mRNA levels of NEK7 in macrophages. Further study demonstrated that A20 directly associates with NEK7, facilitating K48-linked ubiquitination and proteasomal degradation of NEK7. Subsequent research revealed that A20 enhances the ubiquitination of NEK7 at K189 and K293 residues, with K189 being critical for the binding to A20. Moreover, A20 disrupts the interaction between NEK7 and NLRP3 complex, likely through the OTU domain or coordinated function of ZnF4 and ZnF7 motifs [28].

The deubiquitinase YOD1 has been documented to interact with the NACHT and LRR domains of NLRP3, cleaving K33-linked ubiquitination on NLRP3. This process suppresses NLRP3 expression, consequently attenuating inflammasome activation [29]. Ovarian tumor deubiquitinase 6 A (OTUD6A), functioning as a deubiquitinating enzyme, is upregulated in patients with ulcerative colitis and in mice with colitis. Further investigation revealed that OTUD6A directly interacts with the NACHT domain of NLRP3, and selectively hydrolyzes K48-linked polyubiquitin chains at residues K430 and K689, with residue K689 being subject to ubiquitination by SCF-FBXL2 and Cullin1 [30, 31]. This interaction stabilizes NLRP3, augmenting its activity to promote IL-1β production and intensifying inflammatory responses [32]. Ubiquitin specific peptidase 22 (UPS22) and UPS5 suppress NLRP3 inflammasome activation by promoting the autophagic degradation of NLRP3 respectively via ATG5 and the E3 ligase MARCH7 [33, 34]. These studies demonstrate that deubiquitination-associated proteins regulate NLRP3 inflammasome activation primarily by modulating NLRP3 expression and stability during the priming phase.

### SUMOylation and desumoylation of NLRP3 inflammasome

Small ubiquitin-like modifier (SUMO) proteins, part of the ubiquitin-like family, encompass four variants in humans, SUMO1–4. SUMO conjugates to a lysine residue of the substrate and is subsequently cleaved via deSUMOylation mediated by SUMO-specific peptidases. SUMOylation is dynamically coordinated by a dedicated enzymatic cascade: E1 (activating), E2 (conjugating), and E3 (ligase) enzymes mediate SUMO conjugation, whereas sentrin/SUMO-specific proteases (SENPs) catalyze deSUMOylation by cleaving the isopeptide bond to reverse the modification. The SUMOylation cascade is initiated when the E1 enzyme (SAE1/SAE2) covalently links to the C-terminus of SUMO via the sulfhydryl group of a cysteine residue. Subsequently, SUMO is transferred to the E2 enzyme UBC9, and an E3 ligase ultimately directs its conjugation to a lysine residue on the target protein [35]. Enzymes reported to mediate the SUMOylation or deSUMOylation of NLRP3 include TRIM28, mitochondrial-anchored protein ligase (MAPL/MUL1), and SUMO-conjugating enzyme UBC9, which are involved in SUMOylation, as well as SUMO-specific proteases SENP3, SENP6, and SENP7, which catalyze deSUMOylation.

TRIM28 is identified as a potential NLRP3 interactor in LPS-treated mouse primary PMs. Further study revealed that TRIM28 has no effects on *Nlrp3* transcription, but promotes SUMO1- and SUMO2/3-mediated SUMOylation of NLRP3 while suppressing its K48-linked ubiquitination and proteasomal degradation, thereby enhancing the stability of NLRP3 and facilitating inflammasome activation [36]. In the absence of stimulation as well as during LPS priming, the mitochondrial-anchored SUMO E3 ligase MAPL (also known as MUL1) SUMOylates NLRP3 at residue K689 via its RING domain, inhibiting ASC oligomerization and NLRP3 inflammasome activation. While inflammasome-activating stimuli, such as nigericin and ATP, disrupt the interaction between MAPL and NLRP3 and the association between NLRP3 and SUMO2/3, halting MAPL-mediated SUMOylation of NLRP3. Moreover, during this phase, the SUMO deconjugating enzymes SENP6 and SENP7 also promote ASC oligomerization and NLRP3 inflammasome activation [37]. In contrast, UBC9, the sole E2 enzyme for all SUMO proteins, interacts with NLRP3, enabling SUMO1 to catalyze NLRP3 SUMOylation at residue Lys204, finally enhancing ASC oligomerization and inflammasome activation. However, this study does not investigate the E3 ligases involved. Notably, SUMO1 conjugation of NLRP3 in LPS-primed BMDMs shows comparable modification levels to those observed in untreated resting cells, suggesting that SUMO1 modification does not occur during the priming process, but rather is specifically triggered by NLRP3-activating stimuli. In addition, SENP3 indirectly deSUMOylates NLRP3 to attenuate ASC recruitment and speck formation, NLRP3 inflammasome activation, as well as IL-1β cleavage and secretion [38].

These findings manifest that the NLRP3 inflammasome is regulated in a SUMO-dependent manner upon priming and activation stimulation. NLRP3 can be SUMOylated by SUMO1/2/3. SUMO1-mediated SUMOylation promotes NLRP3 inflammasome activation, whereas SUMO2/3-regulated SUMOylation suppresses it. Furthermore, under LPS priming, SUMO2/3 rather than SUMO1 modification functions. Whether SUMO1- and SUMO2/3-mediated SUMOylations of NLRP3 cooperate or act antagonistically to regulate inflammasome activation awaits comprehensive exploration.

### Palmitoylation and depalmitoylation of NLRP3 inflammasome

Protein palmitoylation refers to a reversible lipid modification that is primarily mediated by the zinc-finger and aspartate-histidine-histidine-cysteine proteins (ZDHHCs), which possess a conserved DHHC (Asp-His-His-Cys) cysteine-rich domain that is crucial for enzymatic activity. These ZDHHCs transfer a long-chain fatty acyl group, generally the 16-carbon palmitoyl group, to cysteine residues of target proteins. Growing evidence suggests palmitoylation governs protein trafficking, stability, and intermolecular interactions. Research indicates that NLRP3 palmitoylation is a dynamic process that persists throughout the activation and assembly of this inflammasome, involving both the priming and activation stages [39–42]. Moreover, different ZDHHCs exert varied effects on NLRP3 palmitoylation.

ZDHHCs currently identified as regulators of NLRP3 palmitoylation include ZDHHC12, 5, 7, 17 and 1. ZDHHC12 expression increases in THP1 and DMDMs during the later stages of LPS or ATP treatment, but not in the initial phase, promoting the interaction between NLRP3 and ZDHHC12 as well as NLRP3 palmitoylation in the ER and Golgi apparatus. ZDHHC12 directly catalyzes the palmitoylation of human NLRP3 (hNLRP3) at cysteine residue 844, which enhances its binding to HSC70 and LAMP2A. This interaction facilitates lysosomal degradation via the chaperone-mediated autophagy pathway and consequently suppresses NLRP3 inflammasome activation [43, 44]. However, accumulating evidence indicates that ZDHHC12 has limited involvement in NLRP3 palmitoylation and inflammasome activation [40, 45, 46]. Such discrepancy may arise from the differences in the observed stages. ZDHHC5 is upregulated in THP1 cells during the priming step, which promotes hNLRP3 palmitoylation at Cys837/838 (corresponding to murine Cys834/835) in the LRR domain, thereby enhancing NLRP3-NEK7 interaction and subsequent activation of the NLRP3 inflammasome in response to activation stimuli [43, 45]. ZDHHC7 palmitoylates murine NLRP3 (mNLRP3) at Cys126, promoting the localization of resting NLRP3 on the trans-Golgi network (TGN) during the priming phase and facilitating NLRP3 activation on the dispersed TGN (dTGN) during the activation phase. Subsequently, activated NLRP3 is transported to MTOC, where it mediates the recruitment and oligomerization of ASC, ultimately activating the NLRP3 inflammasome in macrophages. Additionally, this study elucidated the distinct regulatory mechanisms underlying ZDHHC7-catalyzed palmitoylation of mNLRP3 at Cys126 and ZDHHC12-mediated palmitoylation at Cys841, demonstrating that the modification at Cys126 occurs constitutively under basal conditions, whereas palmitoylation at Cys841 is strictly dependent on NLRP3 activation [46]. Emerging evidence establishes that sustained ZDHHC7-dependent palmitoylation of NLRP3 serves as a biochemical prerequisite for its phase separation-driven activation, with this regulatory mechanism being conserved across diverse activation stimuli [47]. Similar to ZDHHC5, ZDHHC17 palmitoylates NLRP3 at Cys419 in the NACHT domain within Golgi upon activation, and promotes NLRP3 activation by interacting with NLRP3 and facilitating NEK7-NLRP3 interactions [48]. ZDHHC1-mediated palmitoylation of hNLRP3 at residues Cys130 and Cys958 (orthologous to murine Cys126/Cys955) regulates its spatiotemporal membrane trafficking toward the MTOC, thereby facilitating LATS1/2-mediated phosphorylation of Ser265 and NEK7 interaction. Specifically, the priming signal-induced palmitoylation of Cys958 enhances the avidity of NLRP3 for its inactive cage structure, thereby stabilizing its localization at the TGN. During early activation, Cys958 palmitoylation promotes the transient anchoring of NLRP3 to the mitochondrial surface. Meanwhile, Cys130-palmitoylated NLRP3 selectively associates with phosphatidylinositol 4-phosphate (PI4P)-enriched membrane compartments, such as the distal TGN and endosomes. This study conducted in human and murine macrophages demonstrates that the palmitoylation modifications of NLRP3 mediates its dynamic subcellular redistribution required for inflammasome assembly [40].

**Fig. 1:**
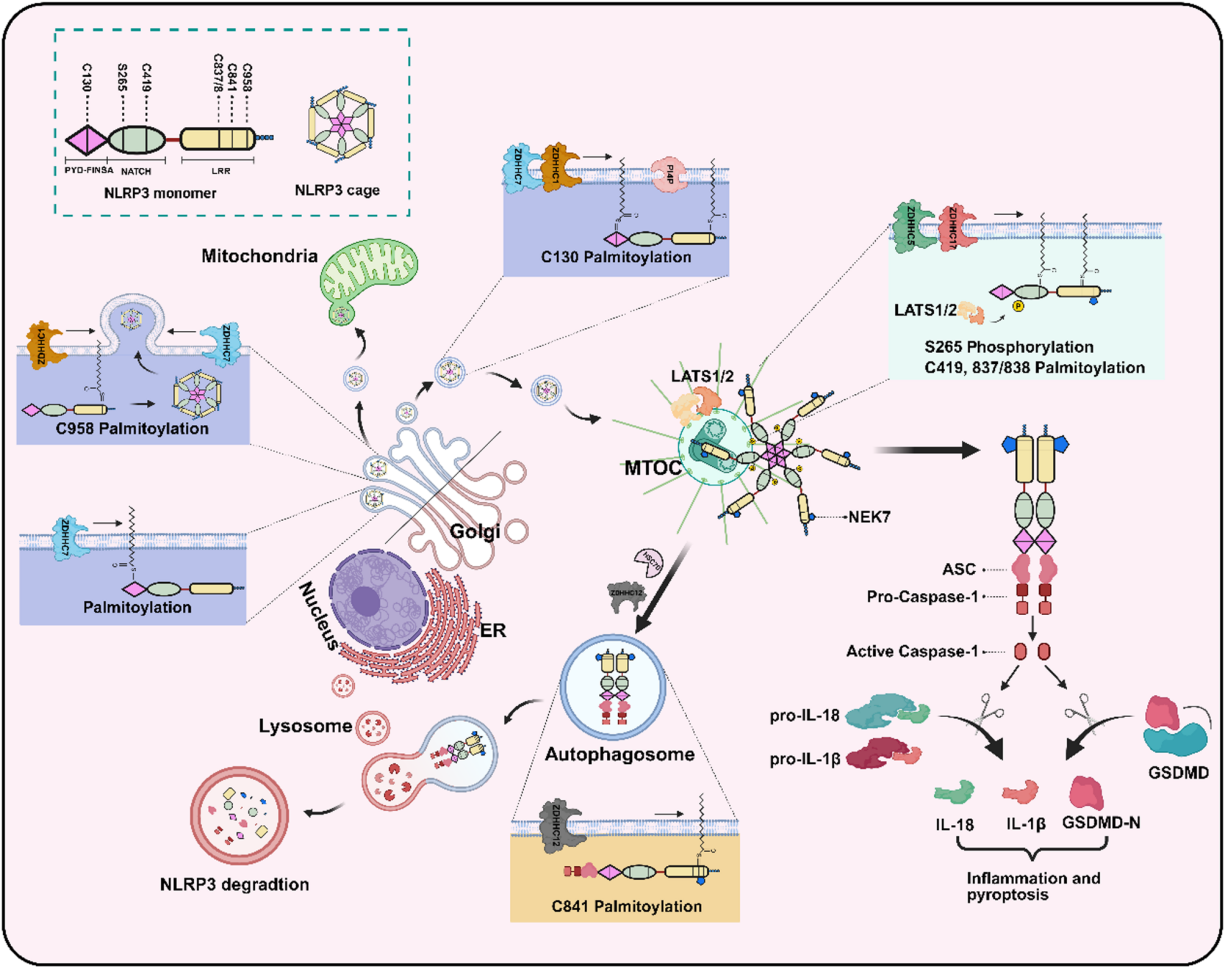
The conjectural regulation processes of palmitoylation on NLRP3. ZDHHC7, 1, 5, 17 and 12 are involved in NLRP3 palmitoylation. Under basal conditions, constitutive ZDHHC7-mediated palmitoylation maintains NLRP3 localization within the trans-Golgi network (TGN). During priming, ZDHHC1-catalyzed palmitoylation at Cys958 promotes NLRP3 oligomerization into double-ring structures while reinforcing its TGN retention. In the early activation phase, Cys958 palmitoylation enables transient mitochondrial translocation of NLRP3, while coordinated modifications by ZDHHC1 and/or ZDHHC7 (via Cys130 palmitoylation) direct NLRP3 trafficking to the dispersed TGN (dTGN) and endosomal compartments. Subsequent transport to the microtubule-organizing center (MTOC) involves ZDHHC5/17-mediated dual palmitoylation at Cys837/838 and Cys419, as well as LATS1/2-dependent Ser265 phosphorylation, which collectively contribute to stabilizing the interaction between NLRP3 and NEK7 and facilitating inflammasome activation. During inflammasome resolution, ZDHHC12 activation induces Cys841 palmitoylation, triggering lysosomal degradation of NLRP3 to terminate inflammatory signaling. Created with BioRender.com

Based on these palmitoylation modifications of NLRP3, we conjecture the following model in Fig. 1. NLRP3 is continuously palmitoylated by ZDHHC7 before the priming step, which leads to resting NLRP3 localization at the TGN. In the priming stage, ZDHHC1-mediated NLRP3 Cys958 palmitoylation promotes the formation of the NLRP3 double-ring cage and facilitates its TGN localization. During early activation, this modification facilitates its transient translocation into mitochondria. ZDHHC1 and/or ZDHHC7-mediated Cys130 palmitoylation ensures NLRP3 localization on the dTGN or endosomes and its transport to the MTOC, where ZDHHC5 and/or ZDHHC17-mediated Cys837/838 and/or Cys419 palmitoylation, along with LATS1/2-regulated NLRP3 Ser265 phosphorylation enhance NLRP3-NEK7 association. In the late stage of inflammasome activation, ZDHHC12 catalyzes Cys841 palmitoylation and brake inflammasome signaling via its degradation in lysosomes. These studies demonstrate that NLRP3 inflammasome signaling is stringently regulated by sequential, site-specific S-palmitoylation events mediated by distinct ZDHHC enzymes, thereby precisely modulating inflammasome activation. Further studies are needed to elucidate how these ZDHHCs are selected and coordinated for NLRP3 inflammasome activation. Currently, several therapeutic strategies targeting ZDHHC12 or the palmitoylation of NLRP3 at Cys126 remarkably inactivate the NLRP3 inflammasome [49, 50].

In addition to these ZDHHCs, protein depalmitoylases, including PPT1, alpha/beta hydrolase domain-containing protein 17 A (ABHD17A), and ABHD13, catalyze the removal of palmitic acid from NLRP3, thereby modulating inflammasome activation. Studies showed a significant phenylpyruvate accumulation in diabetic foot ulcers, which is internalized by macrophages through the CD36 receptor, binds to PPT1 and inhibits its depalmitoylase activity, thereby increasing NLRP3 palmitoylation at Cys6, reducing NLRP3 autophagic degradation, enhancing its protein stability, and promoting inflammasome activation. However, the ZDHHCs responsible for mediating Cys6 palmitoylation remain unidentified [51]. ABHD17A is capable of depalmitoylating NLRP3 at Cys837 and Cys838 to inhibit ZDHHC5-mediated NLRP3 palmitoylation and subsequent inflammasome activation [45]. ABHD13 primarily depalmitoylates NLRP3 at the Cys130 and Cys126 residues, which are palmitoylated by ZDHHC7 [47].

In addition, fatty acid synthase (FASN) plays a crucial role in regulating NLRP3 inflammasome activation, as palmitate is the enzymatic product of FASN and can be reversibly conjugated to cysteine residues of NLRP3, thereby inducing its palmitoylation. Specifically, in LPS-primed mouse BMDMs and human macrophage, TLR ligation induces NLRP3 palmitoylation at Cys898, facilitating its translocation to TGN38-positive vesicles and subsequently priming NLRP3 inflammasome activation upon ATP stimulation [52].

### Acetylation and deacetylation of NLRP3 inflammasome

Protein acetylation is a critical PTM catalyzed by acetyltransferases, which transfer acetyl groups from acetyl donor molecules to specific amino acid residues in target proteins. This modification influences chromatin structure, transcription, and signal transduction, thereby contributing to a wide range of biological processes, including cell cycle, metabolism and so on. In contrast, the histone deacetylase (HDAC) family comprises a group of enzymes that catalyze the removal of acetyl groups from lysine residues on their substrate proteins. HDACs are classified into four distinct classes—class I (HDAC1, 2, 3, and 8), class II (HDAC4, 5, 6, 7, 9, and 10), class III (the sirtuin family), and class IV (HDAC11)—based on their unique structural and functional properties. These acetyltransferases and HDACs respectively mediate NLRP3 acetylation and deacetylation.

Molecules reported to modulate NLRP3 acetylation include Tau, KAT5, and KAT2B. The levels of Tau and pTau proteins are increased at the early stage of Tauopathies. Tau directly catalyzes the acetylation of NLRP3 at the K21, K22, and K24 residues within its PYD domain, independent of nigericin treatment, thereby inducing inflammasome activation [53]. During the assembly signal, KAT5 acetylates NLRP3 at K24, which facilitates NLRP3 oligomerization and inflammasome assembly without affecting its recruitment to dTGN, ultimately resulting in NLRP3 inflammasome activation [54]. KAT2B expression is markedly elevated in high-grade prostate cancer (PCa) tissues compared with that in low-grade PCa and benign prostate hyperplasia (BPH) tissues. KAT2B regulates circAR-3-mediated NLRP3 acetylation, thereby stabilizing the NLRP3 inflammasome complex and promoting its subcellular localization and assembly [55]. These studies demonstrate that NLRP3 acetylation at the PYD domain during Signal 1 contributes to the stabilization of NLRP3, whereas during Signal 2, it enhances oligomerization and inflammasome assembly.

The HDACs involved include HDAC9, HDAC10, SIRT2, and HDAC6. HDAC9 specifically interacts with NLRP3 and facilitates inflammasome assembly through the regulation of acetylation in the NACHT and LRR domains of NLRP3 [56, 57]. HDAC10, a cytosolic deacetylase, directly interacts with NLRP3 and induces deacetylation, which triggers NLRP3 ubiquitination and subsequent proteasome-dependent degradation, thereby suppressing NLRP3 inflammasome activation [58]. The NAD+-dependent deacetylase SIRT2 removes acetyl groups from NLRP3 at residues K21 and K22 that can be acetylated by Tau, thereby inactivating the inflammasome [59]. Notably, HDAC6 facilitates the transport of TGN-localized NLRP3 to the microtubule-organizing center (MTOC), thereby promoting NLRP3-NEK7 interaction and inflammasome assembly. This functional role depends on HDAC6’s ubiquitin-binding domain rather than its deacetylase activity. Further investigation is required to determine which inflammasome components undergo ubiquitination mediated by HDAC6 [60].

### Deglutathionylation of NLRP3 inflammasome

S-glutathionylation is a cellular response to elevated intracellular ROS and is recognized as a redox regulatory mechanism for proteins. To date, no studies have reported glutathionylation of NLRP3. Studies conducted by Hughes et al. have demonstrated that glutathione transferase Omega-1 (GSTO1) regulates NLRP3 inflammasome activation by mediating the deglutathionylation of NEK7 rather than NLRP3 via treating BMDMs with GSTO1 inhibitor C1-27 after LPS priming and before ATP stimulation. NEK7 undergoes deglutathionylation by GSTO1 at Cys253, which enhances NEK7 functions associated with NLRP3 activation. Although GSTO1 interacts with both NEK7 and ASC, it is unlikely to regulate ASC, as ASC is also involved in the activation of AIM2 and NLRC4 inflammasomes, and the GSTO1 inhibitor C1-27 does not suppress their activation [61]. In contrast, another study has demonstrated the critical role of GSTO1 in promoting ASC oligomerization and NLRP3 inflammasome activation through the deglutathionylation of ASC. During the activation phase, GSTO1 interacts with ASC and mediates its deglutathionylation at Cys171 within the ER under the regulation of mitochondrial ROS. Given that the ER serves as the primary intracellular site for Lipid biosynthesis, triacylglyceride levels and its synthetase Diacylglycerol acyltransferase 1 (DGAT1) are determined to be increased. However, DGAT1 deficiency suppresses ASC oligomerization and deglutathionylation without affecting the GSTO1–ASC interaction, suggesting that triacylglyceride synthesis within the ER facilitates GSTO1-mediated ASC deglutathionylation. The mechanisms by which triacylglyceride regulates this process remain to be further elucidated. Additionally, this study reveals that GSTO1 enhances AIM2 and NLRC4 inflammasome activation via mechanisms independent of ASC deglutathionylation [62]. The cell types and stimuli employed in these two studies are highly comparable. Thereby, the discrepancies between the two studies regarding the effects of GSTO1 on AIM2 and NLRC4 inflammasomes may arise from the distinct approaches used to inhibit GSTO1. The former employs a small-molecule inhibitor, C1-27, which selectively and potently inhibits GSTO1 enzymatic activity, whereas the latter applies genetic knockout to suppress GSTO1 expression. Consequently, the conclusion from the latter study — that GSTO1-mediated activation of AIM2 and NLRC4 inflammasomes is independent of ASC deglutathionylation — may be more accurate.

In addition, Caspase-1 deglutathionylation also regulates NLRP3 inflammasome activation. Zhou et al. demonstrated that glutathione S-transferase Mu 1 (GSTM1) specifically deglutathionylates Caspase-1, rather than other components of the NLRP3 inflammasome, ultimately suppressing NLRP3 inflammasome activation [63]. Collectively, these studies indicate that GST-mediated deglutathionylation of NLRP3-interacting proteins or inflammasome components during the activation phase modulate NLRP3 inflammasome activation.

### ISGylation of NLRP3 inflammasome

ISGylation is a type of PTM in which ISG15 covalently attaches to lysine (K) residues of target proteins. This process is mediated by three enzymatic components: the E1 ubiquitin-activating enzyme UBA7 (UBE1L), the E2 conjugating enzyme UBE2L6 (UBCH8), and an E3 ligase—primarily HECT and RCC1 domain-containing protein 5 (HERC5) in humans, along with its murine functional ortholog, HERC6. In LPS-primed mouse primary PMs stimulated with ATP, nigericin, alum, and MSU, NLRP3 undergoes ISGylation by HERC5/6 at lysine residue 799 (Lys799), which is also observed in vesicular stomatitis virus (VSV)-infected or IFNβ-stimulated PMs. This modification competitively inhibits K48-linked ubiquitination at the same lysine residue, thereby preventing proteasomal degradation of NLRP3 and promoting inflammasome activation. Deficiency of *Herc6* ameliorates NLRP3-dependent inflammation as well as hyperinflammation caused by viral infection. This study demonstrates that ISGylation and ubiquitination of NLRP3 converge at a common site, highlighting the dynamic interplay between these PTMs and their reciprocal regulation during inflammasome activation [64].

### S-nitrosylation of NLRP3 inflammasome

S-nitrosylation, a PTM, involves the covalent binding of nitric oxide (NO) to cysteine thiol groups in proteins, resulting in the formation of S-nitrosothiol derivatives. Several proteins, including MAPK14 [65], and muscle LIM protein (MLP) [66], are subject to S-nitrosylation, and modulate NLRP3 inflammasome activation. In addition, NLRP3 itself can also undergo S-nitrosylation by S-nitroso-N-acetylpenicillamine (SNAP), a nitric oxide (NO) donor, thereby inhibiting NLRP3 inflammasome activation in PMs primed with Pam3CSK4 [67, 68]. In addition to the direct S-nitrosylation of NLRP3, NO may also interfere with mitochondrial DNA release and affect other components of the NLRP3 inflammasome, such as Caspase-1 [69]. Therefore, further investigation is necessary to fully elucidate the role of S-nitrosylation as a regulatory mechanism in NLRP3 inflammasome activation.

### Alkylation of NLRP3 inflammasome

As is known, NLRP3 comprises LRR, NACHT, and PYD domains, with the NACHT domain harboring ATPase activity that governs inflammasome assembly and activation through an ADP/ATP switch [70]. Alkylation-mediated inhibition of the ATPase activity of NLRP3 reduces its binding affinity for ATP, thereby suppressing inflammasome activation. Currently reported NLRP3 inflammasome inhibitors, including 4-methylenedioxy-β-nitrostyrene (MNS), 2-cyclohexylimino-6-methyl-6,7- dihydro-5 H-benzo [1, 3] oxathiol-4-one (BOT-4-one), and vanillylacetone, alkylate the cysteine thiol group of NLRP3. This modification subsequently reduces ATP binding, impairs ATPase activity, disrupts NLRP3 self-association and NLRP3-ASC interaction, and ultimately restrains inflammasome activation [71–73]. In addition, itaconate, a bioactive lipid derivative originating from the TCA cycle intermediate cis-aconitate through IRG1/ACOD1-catalyzed decarboxylation [74–76], specifically inhibits NLRP3 inflammasome activation in LPS-primed BMDMs stimulated with ATP or nigericin by alkylating Cys548 and disrupting NLRP3-NEK7 interaction during the activation process [77]. These findings suggest that targeting NLRP3 through alkylation may offer potential therapeutic strategies for NLRP3-related disorders.

## Roles of subcellular compartmentalization in the activation of NLRP3 complex

In addition to the PTMs of NLRP3 inflammasome components, subcellular translocation also contributes to the assembly and activation of the NLRP3 inflammasome [78]. Moreover, the subcellular distribution of NLRP3 is closely associated with PTMs, such as BTK-mediated phosphorylation and ZDHHCs-dependent palmitoylation. NLRP3 has been shown to translocate to various subcellular compartments, including the ER, mitochondria, Golgi apparatus, endosomes, and the MTOC, for its activation. The following section will discuss these organelles and their respective roles in NLRP3 inflammasome assembly and activation.

### Endoplasmic reticulum (ER)

As reported, ER stress and cholesterol are involved in the activation of NLRP3 inflammasome via the NF-κB signaling pathway [79–82]. The ER also serves a platform for NLRP3 inflammasome activation. For instance, during HSV-1 infection and cytosolic DNA stimulation, STING binds to NLRP3 to promote inflammasome activation by both recruiting NLRP3 to the ER and attenuating K48- and K63-linked ubiquitination of NLRP3, which is observed in HeLa cells and THP-1 macrophages [83]. In nigericin or MSU-stimulated mouse BMDMs, dynein-driven microtubule-based transport of mitochondria facilitates the spatial convergence between mitochondrial ASC and ER-localized NLRP3, thereby promoting NLRP3 inflammasome formation [9, 84]. The mitochondria-associated ER membranes (MAMs), which serve as contact sites between mitochondria and the ER, facilitate NLRP3 interaction with oxidized mitochondrial DNA (ox-mtDNA), cardiolipin on the outer mitochondrial membrane, and rapid calcium influx from ER stores into mitochondria, thereby triggering inflammasome activation [79]. However, another study demonstrated that the assembly of NLRP3 inflammasome does not occur at the MAMs. In LPS plus nigericin or ATP-treated mouse BMDMs, MAMs relocate to the vicinity of the Golgi apparatus, where increased diacylglycerol (DAG) facilitate the recruitment and activation of its downstream effector, protein kinase D (PKD). Activated PKD phosphorylates NLRP3 at Ser293, which promotes the dissociation of self-oligomerized NLRP3 from MAMs, thereby enabling its translocation into the cytoplasm and subsequent inflammasome assembly [85]. Disrupting PKD activity retains NLRP3 within Golgi-proximal MAMs, preventing ASC oligomerization and inflammasome activation [86]. Structural studies indicate that, in the unstimulated state, the PYD domain of one NLRP3 molecule closely interacts with the LRR domain of another, leading to the formation of NLRP3 oligomeric complexes on the ER. Upon activation, conformational changes occur within these complexes without resulting in their disassembly [85]. These findings suggest that NLRP3 undergoes oligomerization within the ER, relocates to Golgi-proximal MAMs for PKD-mediated phosphorylation at Ser293, and subsequently recruits ASC in the cytosol to initiate inflammasome activation. However, the precise timing and underlying molecular mechanism of NLRP3 translocation remain to be fully elucidated.

### Mitochondria

In addition to their roles in energy metabolism, mitochondria serve as essential signaling platforms for the assembly and activation of NLRP3 inflammasome. On one hand, mitochondria components, including oxidized mtDNA, mtROS and mitochondrial electron transport chain (ETC), contribute to NLRP3 inflammasome activation [87–89]. On the other hand, mitochondria serve as essential docking sites for the assembly of this inflammasome through interactions involving mitochondrial anti-viral signaling (MAVS) protein, mitofusin-2 (Mfn2), cardiolipin and signal transducer and activator of transcription 3 (STAT3) [90–93]. The role of mtDNA in NLRP3 inflammasome activation remains controversial. Experimental evidence indicates that inflammasome priming and activation are mechanistically linked through inducing new mtDNA synthesis. In LPS and/or ATP-treated mouse BMDMs, TLR4 triggers MyD88/TRIF-dependent interferon regulatory factor 1 (IRF1) activation, upregulating the expression of cytidine monophosphate kinase 2 (CMPK2) to catalyzes the conversion of dCDP to dCTP, thereby providing a critical substrate required for mtDNA synthesis. The newly synthesized mtDNA may be highly susceptible to mtROS and nuclease activity, resulting in the generation of ox-mtDNA fragments that are subsequently released into the cytosol to directly interact with NLRP3 to mediate inflammasome activation [87, 88]. However, other findings indicate that any oxidized DNA regardless of origin or sequence can activate the inflammasome independently of canonical NLRP3 activators, suggesting that NLRP3 May specifically recognize the 8-hydroxy-2’-deoxyguanosine (8-OH-dG) epitopes rather than mtDNA per se [87, 94].

In mouse BMDMs, MAVS recruits NLRP3 to mitochondria via the N-terminal 2–7 region of the PYD in response to stimuli such as poly I: C, ATP, and nigericin, but not crystalline activators like alum, CPPD or MSU, implying that alternative factors may regulate mitochondrial recruitment of NLRP3 under crystalline stimuli. Furthermore, this study demonstrates that NLRP3 oligomerization occurs independently of its mitochondrial recruitment [90]. Upon infection with RNA viruses in LPS-primed BMDMs, NLRP3 and MAVS associate with Mfn2 in a ΔΨ(m)-dependent manner. The HR1 domain of Mfn2 mediates its interaction with NLRP3, thereby facilitating the recruitment of ASC and procaspase-1 to assemble the NLRP3 inflammasome complex [91]. In linezolid-treated BMDMs primed with LPS, NLRP3 inflammasome activation is mediated by sensing mitochondrial dysfunction through the direct binding of its LRR domain to cardiolipin independent of ROS, which subsequently promotes conformational changes that facilitate downstream signaling [47, 92]. In LPS-primed mouse PMs stimulated with ATP, nigericin or MSU, STAT3 binds to NLRP3, undergoes phosphorylation at Ser727 in response to NLRP3 agonists, and subsequently translocates to mitochondria in association with NLRP3 [93]. These findings provide strong evidence that mitochondria serve as a platform for the assembly and activation of the NLRP3 inflammasome following NLRP3 oligomerization. However, it remains unclear whether this docking process occurs at the MAMs.

### Golgi apparatus and endosome

The subcellular localization of NLRP3 is dynamic. Upon nigericin stimulation in LPS-primed THP1 macrophages, the SCAP-SREBP2 complex form a tripartite interaction with NLRP3, facilitating its translocation from the ER to mitochondria-proximal Golgi regions, where inflammasome assembly is spatially optimized [95]. However, Arumugam et al. reported that glycogen synthase kinase 3β (GSK3β) is a molecular determinant for the spatiotemporal regulation of NLRP3 inflammasome activation. Nigericin treatment in HeLa cells initiates GSK3β activation with subsequent binding to NLRP3, facilitating the recruitment of NLRP3 to mitochondria and its subsequent translocation to TGN through transient mitochondria-Golgi contacts. Furthermore, GSK3β-mediated phosphorylation of phosphatidylinositol 4-kinase type II α (PI4K2A) within the TGN promotes NLRP3 translocation to this compartment, thereby enhancing inflammasome assembly and activation [96]. Meanwhile, K^+^ efflux is essential for NLRP3 translocation to TGN. However, once NLRP3 localizes within this compartment, K + efflux is no longer required for the subsequent activation of NLRP3 inflammasome [97]. Chen et al. first demonstrated that different NLRP3 agonists trigger the common formation of dTGN structures derived from the trans-face of Golgi complex. Subsequently, NLRP3 is recruited to the dTGN through ionic bonding between its evolutionarily conserved polybasic (PB) region situated between the PYD and NACHT domains, and the negatively-charged PI4P lipids enriched on dTGN membranes, which promotes NLRP3 aggregation into multiple puncta, as well as inflammasome assembly and activation. These results are primarily obtained in nigericin-treated HeLa cells and validated in ASC-deficient BMDMs [98]. Notably, dTGN formation is dependent on NLRP3 cage-like assembly [99, 100], and may be a prerequisite for initiating NLRP3 recruitment, oligomerization, and activation processes [97, 101]. Follow-up studies have revealed that the NLRP3 PB region alone is insufficient for Golgi recruitment, which requires S-acylation at Cys130 as well as hydrophobic residues located within the α-helical segment (residues 115–125) upstream of this site. Structural Mapping has revealed that a minimal Functional module spanning residues 395 to 699 (NLRP^395–699^) is essential for optimal Golgi localization. However, enhanced Golgi localization does not necessarily correlate with functional inflammasome activation [102]. Moreover, experiments with PB mutants (hNLRP3-3 K/A) demonstrated that NLRP3 PB region is indispensable for recruitment to TGN38 + vesicles, but this region and vesicle recruitment are unnecessary for NLRP3 activation [47, 103]. In iPSC-derived human macrophages, IKKβ recruits NLRP3 to PI4P on the undispersed TGN instead of NEK7, which is sufficient for subsequent inflammasome formation [104].

In these studies, TGN38, commonly employed as a marker of the Golgi, is also expressed in endosomes. Moreover, upon stimulation, the TGN remains largely intact, as confirmed by additional TGN markers. Therefore, vesicles co-expressing NLRP3, EEA1, and TGN38 are of endosome origin rather than TGN or dTGN [105]. Research has found that NLRP3 activators, including K^+^ efflux-dependent nigericin as well as K^+^ efflux-independent imiquimod-derived compound CL097, primarily disrupt ER-endosome membrane contact sites (EECS) in HeLa cells, leading to the accumulation of PI4P in endosomes and consequently impairing endosome-to-TGN trafficking (ETT). This results in the retention of TGN cargo within endosomes, thereby promoting the recruitment of NLRP3 to endosomes and facilitating inflammasome assembly and activation [105]. Other studies have also demonstrated that NLRP3-activating stimuli, including nigericin, lysosomal disrupting agent L-leucyl-L-leucine methyl ester (LLOMe), and imiquimod, can disrupt endosomal trafficking, resulting in the accumulation of PI4P and recruitment of NLRP3 in COS7 cells [106, 107]. Although zinc finger NFX1-type containing 1 (ZNFX1) retains NLRP3 in the cytoplasm and prevents its accumulation in the TGN38+/TGN46 + vesicles and subsequent inflammasome assembly [108], several questions remain regarding the mechanisms by which these activators disrupt EECS, how ETT contributes to endosomal PI4P accumulation, and the process through which NLRP3 is recruited to endosomes.

### Microtubule-organizing center (MTOC)

Centrosomes, the main MTOC, play a critical role in organizing the microtubule network in most animal cells. Mounting evidence demonstrates that the MTOC is involved in NLRP3 inflammasome activation. Furthermore, NEK7, essential for NLRP3 inflammasome activation, localizes to centrosomes [60]. In LPS-primed mouse BMDMs stimulated with ATP, nigericin, alum, or SiO_2_, the centrosomal protein Spata2 suppresses NLRP3 inflammasome activation via recruiting the deubiquitinase CYLD to the centrosome for deubiquitinating polo-like kinase 4 (PLK4), which facilitates the phosphorylation of NEK7 at Ser204, thereby attenuating the interaction between NEK7 and NLRP3 and subsequently inhibiting inflammasome activation [109]. Upon NLRP3 stimuli, including ATP, nigericin, MSU, or alum, microtubule-affinity regulating kinase 4 (MARK4) protein in mouse BMDMs and THP1 cells mediates the delivery of NLRP3 to mitochondria and subsequently to the MTOC, thereby optimizing inflammasome assembly [110]. In NLRP3 stimuli-treated mouse BMDMs, polo-like kinase 1 (PLK1) promotes NLRP3 inflammasome activation during interphase by reinforcing the MTOC structure through γ-tubulin recruitment and microtubule growth, thereby aiding the subcellular positioning of NLRP3 upon activation [111]. HDAC6 also plays a critical role in microtubule-mediated transport and the coordinated assembly of NLRP3 inflammasome. In NLRP3 stimuli-treated mouse BMDMs and THP1 cells, HDAC6 facilitates the directional trafficking of ubiquitinated pathogenic aggregates to the MTOC, where they undergo aggresome formation and subsequent autophagic clearance in lysosomes. These findings reveal that MTOC localization exerts dual activating and inhibiting functions to maintain balanced regulation of NLRP3 inflammasome [60]. However, another study suggests that inflammasome activation may induce the repositioning of the entire Golgi apparatus toward the MTOC, with NLRP3-associated vesicles being trafficked to the MTOC through a microtubule-independent mechanism following TGN dispersion, which is observed in human macrophage using cryogenic fluorescence-guided focused-ion-beam (cryo-FIB) milling and electron cryo-tomography (cryo-ET) [112]. The mechanisms underlying NLRP3 translocation from the Golgi to the MTOC warrant further investigation.

**Fig. 2:**
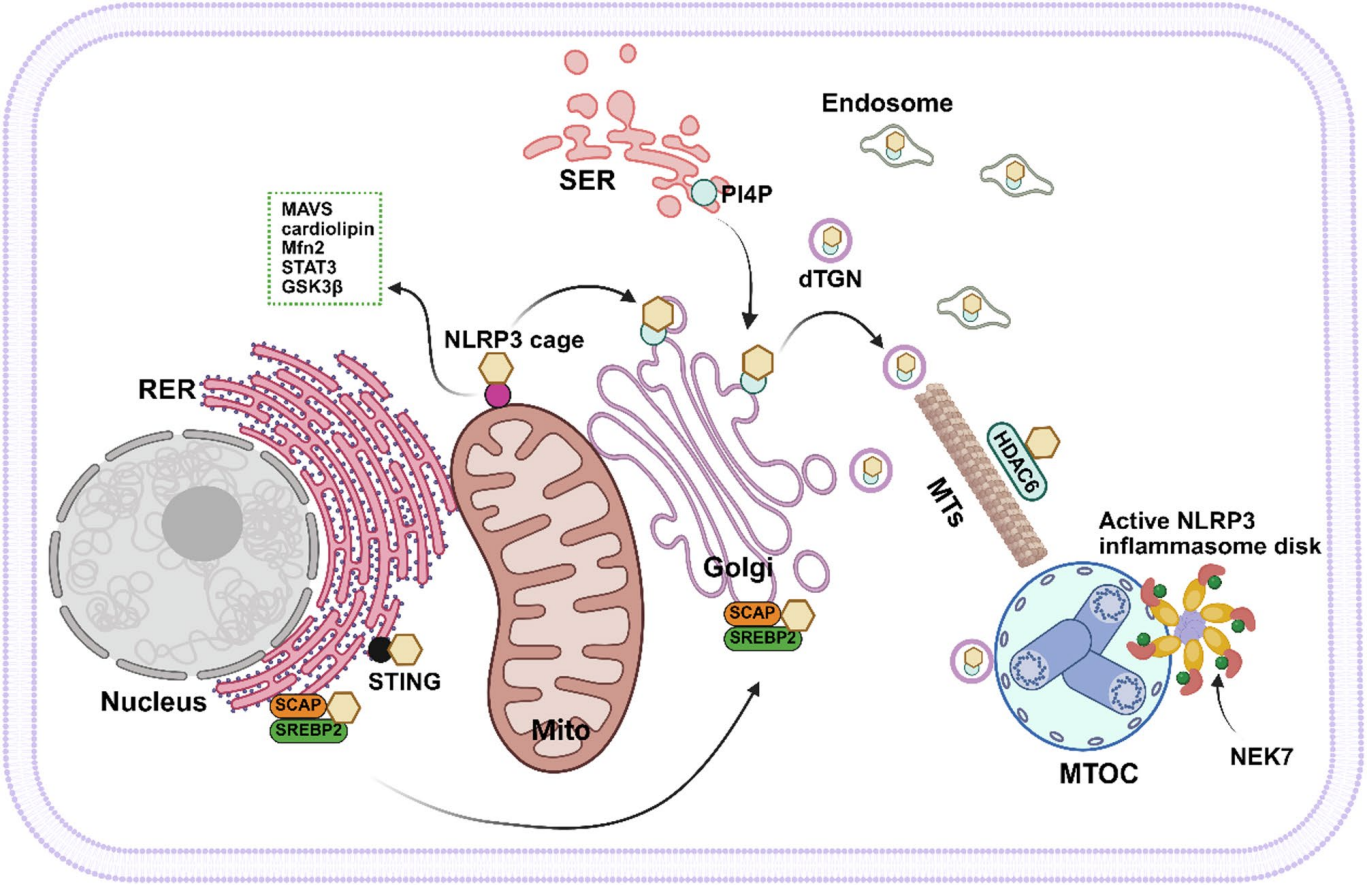
The conjectural spatiotemporal activation of the NLRP3 inflammasome. NLRP3 is oligomerized to form inactive NLRP3 cage in ER, where it interacts with STING or is combined with SCAP-SREBP2 complex that facilitate its translocation to Golgi, finally contributing to inflammasome assembly. NLRP3 cage can be also anchored by MAVS, cardiolipin, Mfn2, STAT3, and GSK3β, which mediate it to the mitochondria for subsequent assembly and activation. PI4P is formed in SER, and then relocated in TGN, dTGN or endosomes, where NLRP3 cage is recruited. Next, NLRP3-associated vesicles are trafficked to the MTOC via a microtubule-dependent or independent mechanism. In the MTOC, NLRP3 cage structure is opened by NEK7 and then binds with NEK7 to activate NLRP3, which facilitates subsequent inflammasome assembly and activation. RER, rough endoplasmic reticulum; SER, smooth endoplasmic reticulum; Mito, mitochondria; dTGN, dispersed *trans*-Golgi network; MTs, microtubules; MTOC, microtubule-organizing center. Created with BioRender.com

These distinct subcellular roles in NLRP3 inflammasome activation may arise from differences in stimuli, cell types, or the focused observation of specific organelles. The ER, mitochondria, Golgi/endosomes and MTOC have been identified as localization sites for NLRP3. However, the temporal sequence and interplay of these localizations remain undefined. During the priming phase, NLRP3 protein is synthesized de novo and translocated to the ER and mitochondrial membranes by fatty acid amide hydrolase (FAAH), which maintains NLRP3 stability by inhibiting its autophagy degradation [113], ultimately positioning NLRP3 in a proper location to rapidly sense inflammatory stimuli. In the activation phase, MAVS, cardiolipin, Mfn2, STAT3, GSK3β, and STING anchor NLRP3 to either mitochondria, ER or both for subsequent assembly and activation [83, 90–93, 96]. Then NLRP3 is translocated from ER or mitochondria to the Golgi apparatus [95, 96], and subsequently from TGN or mitochondria to the MTOC [60, 110]. Cryo–electron microscopy studies of inactive NLRP3 and inflammasome disk suggest a pathway: priming and NLRP3 upregulation, formation of a NLRP3 oligomeric cage in the TGN, TGN dispersion with vesicle-mediated MTOC translocation, NEK7-dependent cage opening, and assembly of the active inflammasome disk [8, 100, 114]. Based on these studies, the conjectural spatiotemporal activation of NLRP3 inflammasome is shown in Fig. 2.

## Conclusion

Currently, significant advances have been achieved in comprehending the molecular mechanisms governing the priming and licensing steps of NLRP3 inflammasome activation. Nevertheless, several pivotal questions remain unresolved, including the mechanisms through which diverse PTMs cooperate to facilitate NLRP3 activation, how distinct activating signals are integrated via common mediators to achieve complete activation of the NLRP3 inflammasome, and the signaling pathways that regulate the final assembly of the inflammasome complex. Moreover, the precise spatiotemporal regulation of NLRP3 inflammasome activation remains to be fully elucidated. However, recent research has broadened the spectrum of PTMs involved in the regulation of NLRP3 inflammasome activity, including (de-)phosphorylation, (de-)ubiquitination, (de-)SUMOylation, (de-)palmitoylation, (de-)acetylation, deglutathionylation, ISGylation, S-nitrosylation, and alkylation, which potentially offer insights into how diverse stimuli initiate inflammasome activation. Additionally, accumulating evidence underscores the involvement of dispersed Golgi apparatus, MTOC, and endosomes in NLRP3 inflammasome activation, alongside the ER and mitochondria. However, the key players responsible for mediating the sequential engagement of organelles and coordinating interorganellar signaling warrant further investigation. Future studies need to comprehensively elucidate the multilayered regulatory mechanisms of NLRP3 inflammasome assembly and activation, which will be critical for establishing a foundational framework for advancing targeted therapeutic and prophylactic strategies against NLRP3-related diseases.

## Data Availability

No datasets were generated or analysed during the current study.

## Abbreviations

NLRP3: The NOD-like receptor pyrin domain-containing 3
PTMs: post-translational modifications
MTOC: the microtubule-organizing center
ASC: apoptosis-associated speck-like protein containing a caspase recruitment domain
PYD: the pyrin domain
NACHT: the nucleotide-binding and oligomerization NAIP, CIITA, HET-E and TEP1 domain
LRR: the leucine-rich repeat domain
NEK7: the Nima-related kinase 7
GSDMD: gasdermin D
TNF: tumor necrosis factor
S1P: sphingosine 1-phosphate
S1PR: S1P receptor
ADP: adenosine diphosphate
NF-κB: nuclear factor κB
BTK: Bruton’s tyrosine kinase
BMDMs: bone marrow–derived macrophages
PP2A: protein phosphatase 2A
PB: polybasic
Ub: Ubiquitin
TRIM24: Tripartite motif-containing 24
PMs: peritoneal macrophages
hESC: human endometrial stromal cells
Insig-1: insulin-induced gene 1
OTUD6A: Ovarian tumor deubiquitinase 6 A
UPS22: Ubiquitin specific peptidase 22
ROS: Reactive oxygen species
SUMO: Small ubiquitin-like modifier
MAPL/MUL1: mitochondrial-anchored protein ligase
SENP3: SUMO-specific protease 3
BMDMs: bone marrow-derived macrophages
ABHD17A: Alpha/beta hydrolase domain-containing protein 17A
HDAC: histone deacetylase
GSTO1: glutathione transferase Omega-1
DGAT1: diacylglycerol acyltransferase 1
TCA: tricarboxylic acid cycle
ER: Endoplasmic reticulum
MAMs: mitochondria-associated ER membranes
ox-mtDNA: oxidized mitochondrial DNA
PKD: protein kinase D
DAG: diacylglycerol
ETC: electron transport chain
Mfn2: mitofusin-2
GSK3β: glycogen synthase kinase 3β
TGN: Trans-Golgi network
PI4K2A: phosphatidylinositol 4-kinase 2 Α
dTGN: dispersed TGN
PI4P: phosphatidylinositol 4-phosphate
EECS: ER-endosome membrane contact sites
ETT: endosome-to-TGN trafficking
PLK4: polo-like kinase 4
MARK4: microtubule-affinity regulating kinase 4
FAAH: fatty acid amide hydrolase
